# Comparison of ALK status between primary and corresponding lymph node metastatic tumors in lung cancer patients

**DOI:** 10.18632/oncotarget.22294

**Published:** 2017-11-06

**Authors:** Qiqi Gao, Honghai Ma, Bo Wang, Yake Yao, Jianya Zhou, Jianying Zhou

**Affiliations:** ^1^ Department of Pathology, The First Affiliated Hospital, Zhejiang University, Hangzhou, China; ^2^ Department of Thoracic Surgery, The First Affiliated Hospital, Zhejiang University, Hangzhou, China; ^3^ Department of Respiration, The First Affiliated Hospital, Zhejiang University, Hangzhou, China

**Keywords:** ALK, metastasis, non-small-cell lung cancer, IHC, FISH

## Abstract

**Background:**

The anaplastic lymphoma kinase (ALK) protein has recently become a promising target in the treatment of non-small cell lung carcinomas(NSCLC) patients with ALK translocation because of the high response rates obtained with an ALK inhibitor. ALK translocations are present in approximately 3-5% of NSCLC patients. According to the literature, little information about the relationship of ALK status between the primary tumor and metastatic sites has been reported. We intended to determine whether the ALK translocations of primary lung cancers are consistent with those in corresponding metastatic lymph node tumors.

**Materials and Methods:**

We analyzed ALK translocations from paired primary and metastatic lymph node tumors in 78 lung cancer patients who had not received TKI before the tissues were sampled.

**Results:**

Using fluorescence *in situ* hybridization (FISH) and immunohistochemistry (IHC) methods, all 45 patients with ALK translocation-positive primary lung tumors had translocations in the metastases. For 33 patients who were ALK negative in the primary tumors, 1 (3%) was ALK positive in their metastatic tumors.

**Conclusion:**

According to a paired t-test, there is no significant difference between primary lung tumors and metastatic sites. Analysis of ALK translocations in the primary lung tumor would be suitable for planning the use of a TKI for advanced NSCLC, but it would be better to detect metastasis specimens as ALK negative specimens if both primary and metastatic specimens have developed.

## INTRODUCTION

Lung cancer is the leading cause of cancer death throughout the developed world. Non-small cell lung cancer (NSCLC) represents the most common histologic subtype of lung cancer cases and is associated with poor prognosis [1]. Almost 70% of patients with NSCLC present with locally advanced or metastatic disease at the time of diagnosis.

Oncogenic ‘driver mutations’ have previously been identified in various subsets of NSCLC [2–4]. NSCLC can be classified into different subtypes according to the oncogenic events that drive carcinogenesis at a molecular level [5]. Epidermal growth factor receptor (EGFR) and anaplastic lymphoma kinase (ALK) are the most common drivers [3, 4, 6]. In 2007, scientists found that anaplastic lymphoma kinase (ALK) gene rearrangements are present in a small subset of non-small cell lung cancers [4]. The overall incidence of ALK gene rearrangement in NSCLC ranges between 0.4% and 13.4%, with no difference between Asian and Western populations [7]. Patients with ALK positive NSCLC tend to be younger and are more likely to be never smokers than those without ALK rearrangement[8].

Crizotinib (an inhibitor of ALK, MET and ROS1) is superior to standard chemotherapy in patients with advanced/metastatic NSCLC that harbors ALK rearrangements in terms of ORR, PFS and QOL by two randomized trials [9, 10]. Based on these studies, crizotinib has been approved for treating patients with NSCLC who express the abnormal ALK gene by the US Food and Drug Administration (FDA) and the European Medicines Agency (EMA). The true therapeutic benefit of molecular targeted therapy in NSCLC patients with ALK fusion protein relies on identifying the right patient population for treatment and detecting the emergence of tumor resistance. ALK rearrangement can be detected by various methods, including immunohistochemistry (IHC)[11, 12], reverse transcriptase polymerase chain reaction (RT-PCR)[13, 14], fluorescence *in situ* hybridization (FISH)[15, 16] and targeted resequencing [17]. IHC and FISH show a good level of correlation [18], and they demonstrated an overall accuracy, specificity and sensitivity of 93%, 95% and 90%[16]. RT-PCR has been recommended in recent international interpretations to resolve discordant or [12]borderline cases [19, 20]. Targeted resequencing proved to be a promising method and means to reduce the material and turnaround time for ALK gene fusion detection. However, there are many challenges before targeted resequences become suitable for an average pathology laboratory [17]. According the Food and Drug Administration (FDA), FISH with the Vysis ALK Break Apart FISH Probe Kit was approved as the gold standard [21].

## RESULTS

### Clinicopathological features of the enrolled patients

The overall clinicopathologic characteristics of the study group are summarized in Table 1. Seventy-eight patients were enrolled into this study with a median age of 58 years old (range, 29 - 77 years). The seventy-eight patients had an average age at diagnosis of their primary lung carcinoma of 56 years. Among them, 43 (55.1%) were females, and 60 (76.9%) were non-smokers. The primary diagnosis was made in lobectomy (76 cases; 97.4%) and wedge resection (2 cases; 2.6%) specimens. The histologic subtype of the primary lung carcinomas was predominantly adenocarcinomas (76 cases, 97.4%), including solid adenocarcinoma (29 cases, 37.2%), papillary adenocarcinoma (16 cases, 20.5%), mucinous adenocarcinoma (15 cases, 19.2%), acinar adenocarcinoma (15 cases, 19.2%) and micropapillary adenocarcinoma (1 case, 1.3%). The 2 other histologic types of NSCLC included a case of adenosquamous carcinoma and a case of squamous carcinoma. Forty-seven of the primary tumors (60.3%) were located in the right lung and 31 (39.7%) were in the left lung. The size of the primary tumors ranged from 1.5 to 9 cm. All of the metastatic tumors were synchronous metastases of lymph nodes and identified in surgical pathology material.

**Table 1:** Clinicopathological features of the 78 enrolled patients

Classification	Characteristics	n	Alk rearrangement	P
Location	Primary site	78	45	0.871
	Paired metastatic lymph	78	46	
Age years	Median	56	53.7	0.894
	Range	29-77	29-77	
Sex	Male	35	19	0.494
	Female	43	27	
Smoking	Never smoker	60	38	0.153
	Former and current smoker	18	8	
Predomina nt pattern	Solid	29	22	<0.05
	Acinar	15	4	
	papillary	16	5	
	Mucinous	15	12	
	Micropapillary	1	1	
	adenosquamous carcinoma	1	1	
	squamous carcinoma	1	1	

For continued treatment after surgery of the NSCLC patients with ALK positive primary tumors, 12 patients received targeted agents, and all of them survived; 21 patients received chemotherapy, 4 of them died, and 17 of them survived. For the continued treatment after surgery of the NSCLC patients with ALK negative primary tumors, 23 patients received further treatment (including chemotherapy and targeted agents), and 2 patients died; 19 patients survived, including the patients with ALK positive metastatic tumors, and 2 patients were lost to follow up; 7 patients did not receive further treatment, 3 patients survived, and 4 patients were lost to follow up; 3 patients received unknown treatment and died.

**Figure 1:**
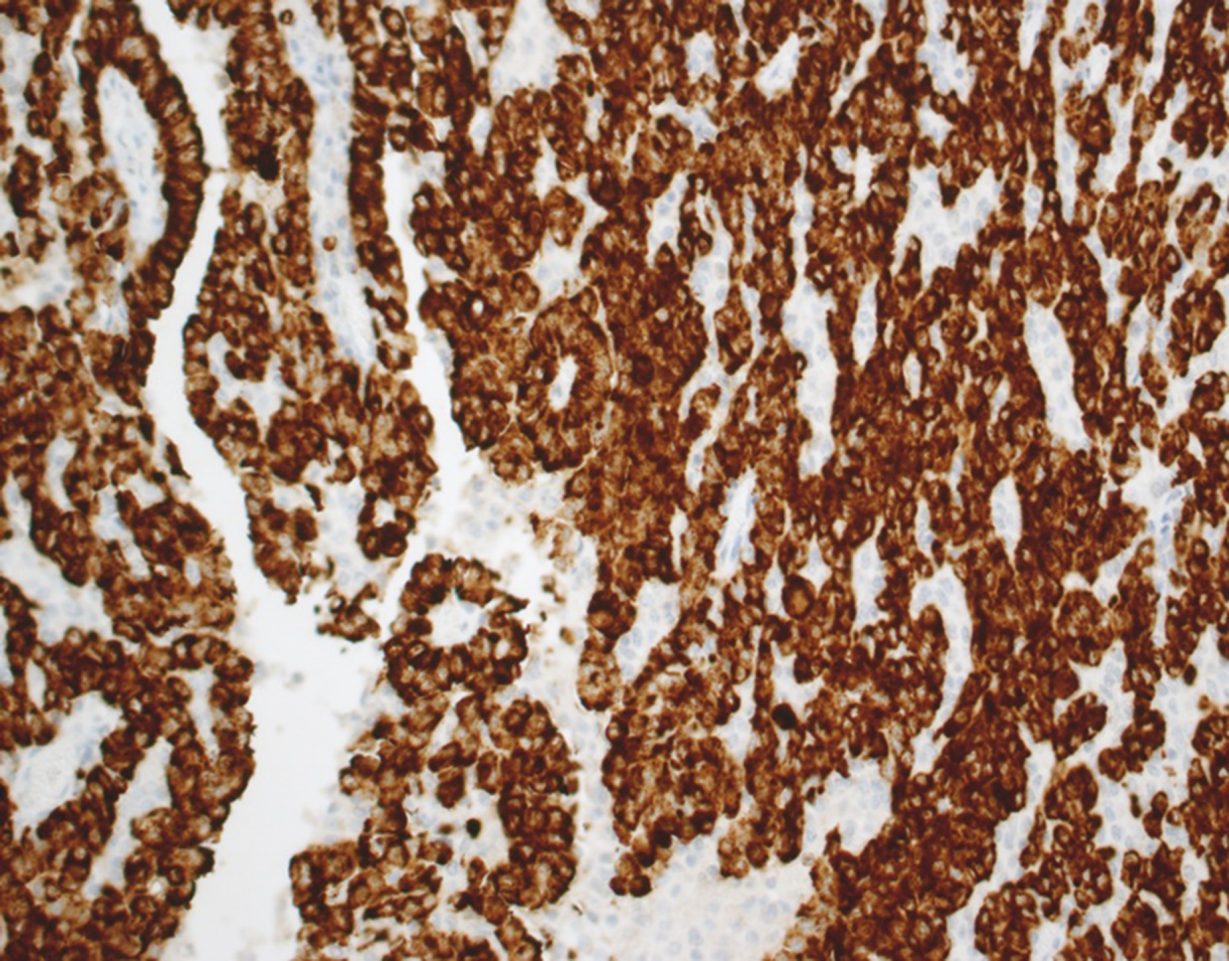
Immunohistochemistry for ALK in a NSCLC Tumor cells show cytoplasmic expression of the protein.

**Figure 2:**
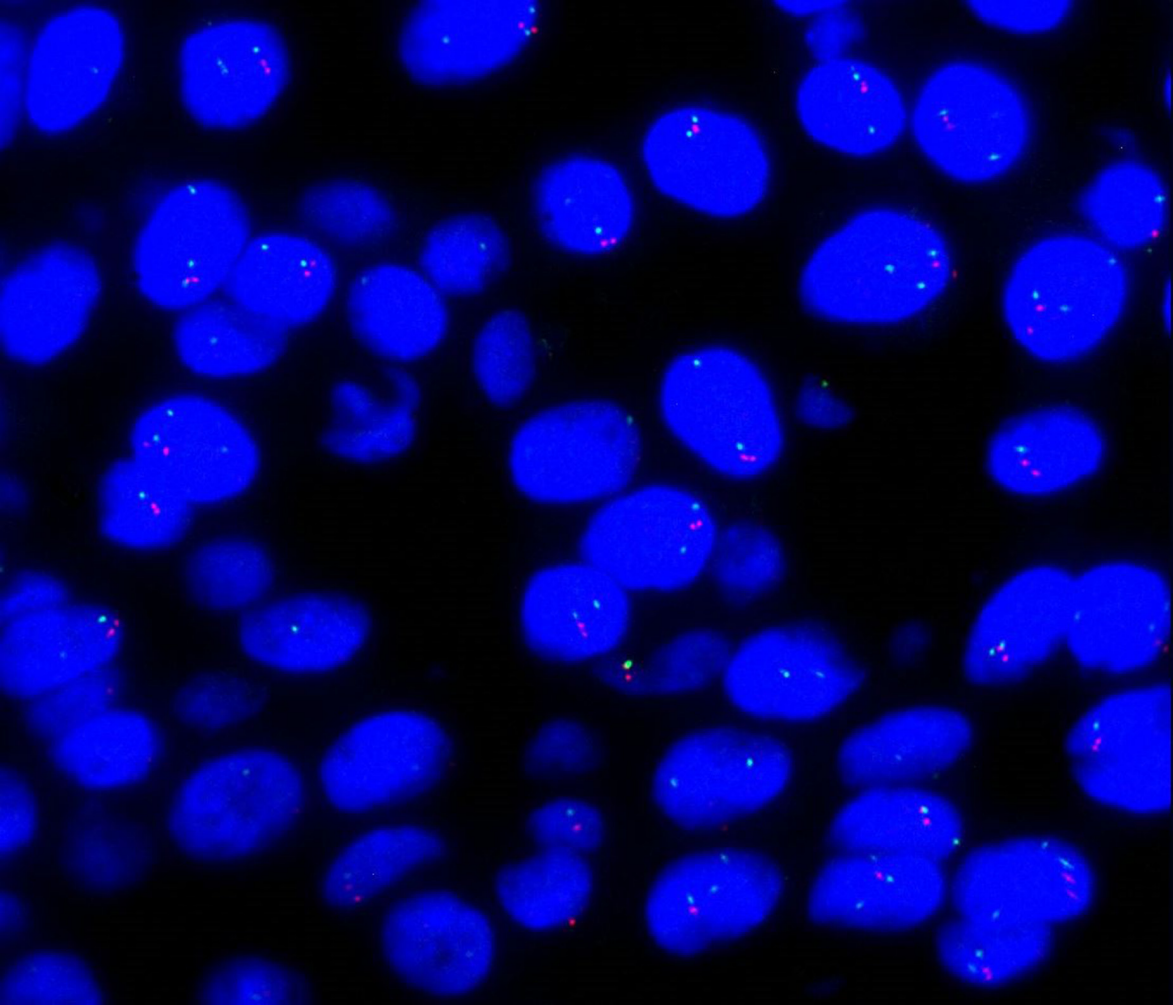
FISH analysis Positive cells show a fusion of the red and green signals corresponding to the intact chromosome, and the split signals are indicative of the ALK rearrangement.

The concordance rate of ALK rearrangement between primary tumor and paired metastatic lymph nodes was detected by immunohistochemistry (IHC) and FISH. The ALK gene was evaluated using IHC and FISH in 78 paired specimens (primary tumor site and paired metastatic lymph nodes). Of the 78 paired specimens detected by IHC and FISH, 32 paired specimens were both IHC and FISH negative, and 45 paired specimens were both IHC (Figure 1) and FISH (Figure 2) positive (as shown in Table 2). One (1.3%) patient showed ALK amplification in the metastasis but not in the primary sample. Seventy-seven (98.7%) patients showed concordant ALK rearrangement between primary tumors and paired metastatic lymph nodes.

**Table 2:** *ALK* rearrangement concordance between primary tumor and paired metastatic lymph nodes by IHC and FISH (77 of 78 cases)

Methods	Primary site (No.)	Paired metastatic lymph nodes (No.)
IHC−	33	32
IHC+	45	46
FISH-	33	32
FISH+	45	46

## DISCUSSION

NSCLC represents the leading cause of cancer death throughout the developed world, but the introduction of a novel class of targeted anti-neoplastic agents, EGFR and ALK TKI directed against EGFR and ALK, has significantly changed the therapeutic options available for patients with NSCLC. ALK gene rearrangements are found in 2–7% of all NSCLC patients and are more likely to be found in younger patients (<50 years old) and light/never smokers and have an adenocarcinoma histology, predominantly the signet ring cell subtype [22]. According to the literature, ALK gene rearrangements have a relatively higher frequency in Asian populations and were reported as high as 5.1–10%[23–26]. The incidence of ALK rearrangement was 19.8% in patients with wild-type EGFR [27]. ALK translocation has become a promising candidate for a therapeutic target as well as a diagnostic molecular marker described for NSCLC cancer patients [4] due to the excellent responses observed in lung tumors with the ALK rearrangement treated with ALK inhibitors [28].

Knowledge of the clinical implications of ALK rearrangement has been rapidly generated; however, there are still problems that remain unclear, such as whether ALK translocations were concordant between primary tumors and their associated metastases. Intratumor heterogeneity has been recently described, and it is a major challenge for personalized cancer medicine. Previous studies have compared the molecular state between primary tumors and corresponding metastatic tumors. We have learned from other biomarkers that genetic variation present in the primary tumor may not exist at the metastatic site, and vice versa. The studies mainly focused on EGFR and related biomarkers in lung cancers in the studies comparing the molecular status between primary cancers and matched metastases. KRAS mutations and EGFR FISH results differ between primary lung tumors and their corresponding synchronous or metachronous metastases [29]. Low concordance of KRAS mutational status exists between the primary tumors and paired metastasis [30]. However, according other studies, there was high concordance between the primary tumors and paired metastasis. Lee WS et al. indicated that both primary lesions and their corresponding metastases showed the same level and pattern of HER2 expression [31]. However, few studies about intratumor heterogeneity of ALK rearrangement have been performed. Rossi A et al. published a case of a lung cancer patient with pleural and peritoneal metastasis, whose pleural metastases were ALK negative, while their peritoneal metastases were ALK positive [32]. However, another case report found ALK was positive in both primary and metastatic lesions [33] and, Likun Hou et al. demonstrates a high concordance rate (98%) in the ALK rearrangement between primary tumors and paired metastatic lymph nodes [27]. Our results are consistent with this report and suggest that cancer cells remain fairly stable in metastatic tumors. According to previous studies, ALK rearrangement in the primary tumor of two patients did not show ALK fusion gene on paired metastatic lymph nodes [27]. In our study, we found that all primary tumors with ALK rearrangement were also positive in matched lymph node metastases, and all primary tumors without ALK rearrangement were negative in matched lymph node metastases, except one patient who was ALK rearrangement negative in the primary tumor while positive in the matched lymph node metastases.

A true gold standard to determine ALK has not yet been established. In previous studies, IHC technique and various molecular techniques, including reverse transcriptase polymerase chain reaction, direct sequencing, and FISH, have been used. FISH was approved as a guide diagnostic by the Food and Drug Administration. Some studies have reported that in clinical practice, Ventana IHC is a valuable tool for screening patients with ALK rearrangements [16, 19, 34]. Yi ES et al suggested that all IHC 3+ cases were FISH positive, whereas 1 of 3 IHC 2+ and 1 of 21 IHC 1+ cases were FISH positive. All 69 IHC 0+ cases were FISH negative. We screened patients with IHC 3+ or 0+ primary lesions, removing the patients with 1+ or 2+ primary lesions. In our case, all IHC3+ lesions were FISH positive, and IHC 0+ were FISH negative, the same as previously reported [35].

In summary, we have shown that ALK rearrangement in primary lung tumors always reflects the same situation in metastases. Therefore, ALK-targeted TKIs can be used to treat metastatic disease.

## MATERIALS AND METHODS

### Patient selection and tumor samples

Patients were selected from a pathological database of lung cancer cases undergoing surgical resection of the primary tumors and the corresponding lymph node metastatic sites at the Pathology Department of The First Affiliated Hospital of Zhejiang University, and the primary and paired metastatic lymphatic section had to contain a sufficient number of tumor cells. The cases with inadequate tumor tissue in primary or metastatic tumors were excluded. The enrollment criteria included primary sections with IHC ALK 3+ from September 2013 to January 2016; only 45 patients were selected among 488 patients. Thirty-three patients with primary sections with ALK 0+ among 128 patients were also enrolled from January 2015 to May 2015. Consequently, a total of 78 pairs of formalin-fixed, paraffin-embedded surgically resected lung cancer tissues and corresponding resected metastatic tumors were analyzed.

All of the patients were examined for age, gender and smoking status, partly for cancer stage and treatment. Lung cancer histology was defined according to the World Health Organization pathology classification [36]. Clinicopathologic staging was defined according to the International Union against Cancer tumor–node–metastasis classification of malignant tumors. Clinical pathological characteristics of the 78 cases were listed in Table 1. None of the patients received systemic chemotherapy or chest radiotherapy before the operation. None of the patients were treated with a TKI.

### Tissue microarray

Tissue microarrays (TMAs) were constructed from tissue blocks used for routine pathological evaluation. All original archived hematoxylin-eosin–stained slides were individually reviewed. Areas in each case with the most representative histology were selected and marked on individual paraffin blocks. The most representative tissue core was obtained from each tumor specimen and extruded into the recipient array. A section from each microarray was stained with hematoxylin and eosin and examined by light microscopy to assess the adequacy of tissue sampling. Tissue microarray (TMA) specimens were assembled by a TMA instrument (TMA Master; Chaoyang, China), which contained thin-walled stainless steel punches and stylets for emptying and transferring the needle apparatus. The instrument was used to create holes in recipient blocks with defined array cores. A fit needle was used to deliver the tissue cores into the recipient blocks. The percentage of tissue core taken from within the tumor was >70%.

### ALK protein expression

Immunohistochemical staining was performed on a Ventana BenchMark XT automated slide-processing system at Ventana Medical Systems. Immunohistochemical staining was performed using 2-μm sections of TMA blocks. All slides were stained with ALK antibody (clone D5F3 Cell Signaling Technology) diluted 1:50 using a Ventana Ultraview DAB detection kit in a Ventana BenchMark XT processor (Ventana Medical Systems, Inc, Tucson, AZ). Antigen retrieval was a standard automated process on the Ventana BenchMark XT at 37°C for 16 minutes. All immunohistochemical stains were evaluated independently by two pathologists. Specimens were scored 3+ if strong granular cytoplasmic brown staining in tumor cells was present (excluding non-tumor cells: alveolar macrophages, cells of neural origin, glandular epithelial staining), and the absence of cytoplasmic staining was classified a negative result as described previously [37].

### ALK fluorescence *in situ* hybridization (FISH) analysis

The Break-apart Rearrangement Probe kit (Abbott Molecular, Abbott Park, IL) was used for FISH. We prepared 4 mm paraffin-embedded histological sections for FISH analysis. Tissue sections were baked at 60°C and deparaffinized, and then pretreated with protease solution at 37°C for 20 minutes. The slides were denatured at 75°C (±2°C) for 10 minutes. ThermoBrite (Abbott Molecular, IL, USA) was used for hybridization at 37°C overnight for 18 hours. Nuclear counterstaining with DAPI was performed after stringency wash steps. Sections were observed under a ×100 objective with a fluorescence Leica microscope and microsystem Imaging system (Leica Microsystems Inc., Buffalo Grove, IL) independently by two experienced pathologists. A minimum of 100 nuclei was scored. A FISH positive case was defined as having more than 15% (≥15/100) tumor cells showing separated green and red signals (at least two signal diameters apart) or single red signals (deleted green signal) identified cells with rearranged ALK.

### Statistical analysis

The difference in demographic characteristics, including age, gender, smoking history, pathological type, and TNM stage between ALK positive and ALK negative patients, were analyzed by Pearson chi square test and Fisher test. Differences were considered statistically significant when the two-sided P-value was ≤0.05. SPSS software (version 19.0, Chicago, IL, USA) was used for statistical calculation.
